# Cancer immunoprevention: from mice to early clinical trials

**DOI:** 10.1186/s12865-018-0253-0

**Published:** 2018-06-15

**Authors:** Arianna Palladini, Lorena Landuzzi, Pier-Luigi Lollini, Patrizia Nanni

**Affiliations:** 1 Department of Experimental, Diagnostic and Specialty Medicine (DIMES), University of Bologna, Viale Filopanti 22, 40126 Bologna, Italy; 2 Laboratory of Experimental Oncology, Rizzoli Orthopaedic Institute, Via di Barbiano 1/10, 40136 Bologna, Italy

**Keywords:** Cancer immunoprevention, Cancer vaccines, Genetically-modified mouse models, HER-2, Oncoantigens

## Abstract

Cancer immunoprevention is based on the fact that a functioning immune system controls tumor onset and development in humans and animals, thus leading to the idea that the enhancement of immune responses in healthy individuals could effectively reduce cancer risk later in life. Successful primary immunoprevention of tumors caused by hepatitis B and papilloma viruses is already implemented at the population level with specific vaccines. The immunoprevention of human tumors unrelated to infectious agents is an outstanding challenge. Proof-of-principle preclinical studies in genetically-modified or in carcinogen-exposed mice clearly demonstrated that vaccines and other immunological treatments induce host immune responses that effectively control tumor onset and progression, eventually resulting in cancer prevention. While a straightforward translation to healthy humans is currently unfeasible, a number of pioneering clinical trials showed that cancer immunoprevention can be effectively implemented in human cohorts affected by specific cancer risks, such as preneoplastic/early neoplastic lesions. Future developments will see the implementation of cancer immunoprevention in a wider range of conditions at risk of tumor development, such as the exposure to known carcinogens and genetic predispositions.

## Background

The immune system is a major player in the prevention of diseases. Immunity is best known for the prevention of infectious diseases, however it plays an equally important role in the prevention of tumors. Such a role was first hypothesized half a century ago, but a definitive demonstration came only at the beginning of this century, when it was shown that severely immunodeficient mice invariably develop tumors over time, whereas immunocompetent mice of the same age are tumor-free [1, 2].

While mouse immunologists were struggling to devise appropriate genetically-modified immunodeficient mouse models, human immunologists accumulated evidence on transplant recipients and AIDS patients, showing that, in both cases, human immunodeficiency brought about a strong increase in the risk of virus-related tumors, such as Kaposi sarcoma, caused by human herpesvirus 8, or carcinomas caused by human papilloma viruses [3].

After 50 years of intense research, we have reached general conclusions that apply to humans and mice: any severe, congenital immune deficiency exposes the adult host to a high risk of tumor onset involving all tumor types. Partial or transient immune deficiencies entail a correspondingly reduced tumor risk, possibly limited to specific tumor types, such as highly immunogenic viral tumors [4]. Under non-sterile conditions, severe primary immune deficiencies expose the host to an early septic death, well before the age when the tumor risk would become manifest, hence human cancer risk related to immune deficiency is mostly confined to secondary immune deficiencies and viral tumors [5].

Just as it happens with infectious diseases, the tumor preventive efficacy of the immune system is far from complete and declines with age, thus contributing to the age-related risk of cancer [6]. From a preventive point of view, this leads to the concept of cancer immunoprevention, i.e. the opportunity to further decrease tumor risk through the stimulation of immune defenses [7, 8].

## Immunoprevention of viral and non-viral tumors

Human cancer immunoprevention is clearly divided in two: on the one hand are tumors related to infectious agents, for which some effective vaccines are already implemented at the population level, even though some difficulties remain, as we shall see. On the other hand are all tumors unrelated to infectious agents (here referred to as noninfectious tumors), which represent the bulk of human tumor burden [9], for which we are beginning to see some early clinical application, after two decades of tantalizing preclinical results in mouse models [10, 11].

One issue that must be clarified in advance is that the notion of cancer prevention, and by extension of cancer immunoprevention, encompasses conceptually different approaches (*see* [12] for a more formal definition of the various types of prevention). *Primary prevention* aims at removing cancer risk factors to reduce tumor incidence. A classic example in the field of chemical carcinogenesis is the avoidance of cigarette smoke, to prevent the onset of lung cancer and many other tobacco-related tumors. Vaccines against oncogenic viruses are a typical example of primary cancer immunoprevention. In the field of primary cancer prevention, the use of drugs that reduce the risk of cancer, for example by preventing exposure to carcinogenic agents, is labeled as chemoprevention. Given that vaccines are drugs, immunoprevention can be also defined as a type of chemoprevention that acts through the immune system [13]. *Secondary prevention* aims at limiting cancer progression toward malignancy, through interventions targeted at early stages of tumor onset. Early diagnosis is the classic human application labeled as secondary prevention, implemented through mass screenings, for example using mammography. However, it must be considered that early diagnosis is only the beginning of secondary prevention, and an early therapeutic intervention is needed to actually avoid progression to malignancy. Thus, immunological treatments applied after an early diagnosis, to prevent tumor progression, are instances of secondary cancer immunoprevention [12].

When one considers that the carcinogenic process is a continuum that goes from a normal tissue to a highly malignant tumor, in some instances it is a matter of definition whether a given intervention should be labeled as primary or secondary prevention. From a practical perspective this can result either in the reduction of incident tumors, through the discovery of lesions labeled as preneoplastic, or in the increase in the number of early neoplastic lesions, eventually producing a higher number of tumor diagnoses. From the point of view of secondary prevention, tumor incidence is not an issue, because what really counts is the decrease in cancer mortality. In this review we will adopt a more conceptual perspective, and we will consider as secondary prevention any intervention taking place after the start of the carcinogenic process, regardless of whether the underlying abnormal tissue is formally labeled as preneoplastic or as early neoplastic.

## Prevention of infection-related tumors

Outside of the laboratory, the first successful application of vaccines to the prevention of cancer was in the late 1960s to Marek’s disease, an avian leucosis that affected poultry farms, caused by the eponymous herpesvirus MDV [14].

The first human cancer preventive vaccine was against hepatitis B virus (HBV), which in a minority of infected individuals could result in chronic hepatitis, hepatic cirrhosis and eventually hepatocellular carcinoma. The earliest confirmation of the tumor preventive activity of anti-HBV vaccines came from a pediatric Taiwanese cohort, which showed a 70% overall reduction of liver cancer risk, further confirmed in a subsequent long-term re-evaluation [15].

Worldwide implementation of HBV vaccination programs in the 1980s thus represents the first instance of mass cancer immunoprevention. It might be objected that prevention of acute HBV infection is the *raison d’être* of the HBV vaccine, and that cancer prevention is just a nice, but secondary, side effect. The point is well taken, but certainly it does not apply to the second wave of cancer preventive vaccines, directed against human papillomaviruses (HPV), which are essentially oncogenic viruses [16], hence any HPV vaccine is by definition aimed at cancer prevention.

Mass vaccination against HPV begun in the late 2000s, thus long-term results for what concerns cancer prevention at the population level are not yet available, but the results of approval trials, which involved tens of thousands of women worldwide, showed near 100% prevention of neoplastic lesions caused by the viral genotypes included in each vaccine [17]. Furthermore, early analyses of national vaccination programs confirm sizeable reductions in HPV prevalence [18], foreboding corresponding reductions in cancer incidence.

In principle, cervical carcinoma could become the first human cancer eradicated by immunoprevention, much as it happened with smallpox in the late 1970s. However, major obstacles must be overcome before this happens. The major one is that in most countries the proportion of subjects vaccinated each year is low, even down to less than 50% in some US states [19]. The reasons of this phenomenon are beyond the scope of this review, but it is clear that, unless the worldwide level of population compliance rises significantly, the hope to eradicate cervical carcinoma through immunoprevention will remain in the realm of dreams.

We still don’t have vaccines approved for two major cancer-related infectious agents, hepatitis C virus (HCV) and *Helicobacter pylori*, however there are highly efficacious drugs that can eradicate both, effectively preventing HCV-related hepatocellular carcinoma and gastric cancer (the efficacy of such drugs also hampers the development of vaccines, but again this is a subject beyond the scope of this review). Altogether, we have in our hands the potential to prevent the vast majority of infection-related cancers, which represent about one sixth of the total human cancer burden [9].

## Prevention of non-infectious tumors

Conversely, about 85% of all human tumors are unrelated to infectious agents [9]. What are the perspectives of immunoprevention in such cases?

If one looks at animal models of cancer, the problem is already solved: many researchers, including ourselves, have demonstrated time and again that a variety of immunological treatments, administered to healthy, cancer-prone mice, effectively prevent the onset of tumors later in life [13, 20]. So, what are the obstacles to an immediate translation to humans of the results obtained in mice?

The dirty little trick in all animal studies of cancer immunoprevention is that the researchers know *in advance* which type of tumor will arise in their mice, and when it will appear, whereas humans are exposed to the risk of many different tumor types over several decades of their life. Basically, this means that in the near future we are not going to have a generic “vaccine against cancer” to be administered to all children to reduce their lifetime risk of cancer. However, there are several human cohorts subject to a predictable and measurable risk of a known tumor, who could greatly benefit from the implementation of specific vaccination programs.

In the following sections we will first summarize the results of preclinical studies, then we will examine some examples of the earliest clinical trials of cancer immunoprevention.

## Cancer immunoprevention in mice

The two major types of mouse models used to investigate cancer immunoprevention are conventional mice treated with carcinogens and cancer-prone genetically-modified mice [13, 20]. Most studies in the past 20 years used genetically-modified mice, mirroring the generalized success of these model systems in all fields of biomedical research [21]. The standard experiment sees young, tumor-free mice undergoing immunological maneuvers that delay tumor onset later in life, or result in a lower incidence of tumors.

Positive results were obtained against a myriad of cancer types, using either passive approaches, e.g. administration of monoclonal antibodies, or active stimuli, which in turn included antigen-specific vaccines, or non-antigen-specific treatments, such as cytokines or other immunostimulants [2, 13, 20, 22]. It could be concluded that, in mice prone to cancer, immunoprevention is generally doable and is not dependent on model-specific or treatment-specific experimental conditions [13, 20, 23].

The analysis of protective immunological responses revealed some differences with those elicited by vaccines used in cancer immunotherapy, which are mainly focused on cytotoxic T lymphocytes (CTL). Many studies showed a relevant role for helper T cells, B cells and their products, i.e. cytokines and antibodies [24]. A major determinant could be the different time frame of immunoprevention in comparison to immunotherapy. In fact, immunoprevention entails immune responses that must be ideally active during the entire life of the host, and mouse experiments typically last from several months to more than one year. Under these conditions a prolonged CTL response would probably produce relevant toxic side effects, whereas the humoral response can persist indefinitely at protective levels without harm for the host. A similar dichotomy is encountered in viral immunity: in most instances the cure of acute infection requires the CTL response, whereas natural prevention of reinfection and vaccine efficacy are mainly dependent on antibodies [25].

## Target antigens

A specific aspect of cancer immunoprevention in relation to cancer immunotherapy is the choice of target antigens [26]. In the field of cancer immunotherapy there is currently much interest for neoantigens, i.e. random alterations of normal molecules resulting from the carcinogenic processes [27]. However the intrinsically unpredictable nature of neoantigens makes them unsuitable as targets in cancer immunoprevention. It has been argued that the best targets for cancer immunoprevention are oncoantigens, i.e. those molecules that are causally involved in the carcinogenic process, because their inhibition in preneoplastic lesions or in early tumors offers the opportunity to block tumor progression and minimizes the emergence of antigen-loss variants [2, 28–30].

The ideal target antigen for cancer immunoprevention would be a molecule expressed only by neoplastic or preneoplastic cells, however only a few molecules fulfill this requirement, such as MUC1, which is differentially glycosylated in normal and neoplastic cells [31, 32], or HPV-encoded molecules in HPV-infected people. In most instances the target antigen would be expressed also by some normal cells. In this case an important issue is the physiological role of the target antigen, because (auto)immune responses directed against targets that play a relevant role in the biology of the healthy adult host are bound to provoke intolerable toxicities. Under this respect, the HER-2 oncogene, which was extensively studied both in mice and in humans, is a good choice, because its most important physiological role appears to be during heart embryogenesis [33], whereas long-term inhibition in adults, as it happens during monoclonal antibody therapy of breast cancer, is well tolerated in the vast majority of patients [34]. Such antigens would traditionally be labeled as oncofetal antigens, but more recently the term “retired antigens” has been proposed in relation to cancer immunoprevention [35].

## Early clinical trials

We will examine here some early clinical trials demonstrating that cancer immunoprevention is indeed translatable to appropriate human contexts. A detailed discussion of the countless clinical trials in which vaccines were tested as therapeutic agents against established human tumors goes beyond the scope of this review, in particular because most therapeutic cancer vaccines of the past had limited efficacy against existing human tumors [20, 36]. A renaissance of therapeutic cancer vaccines is currently being fostered by the molecular definition of novel antigens (neoantigens) appearing in individual tumors as a consequence of extensive mutational events in the genome (“mutanome”) [37]. New therapeutic vaccines are also being combined with immunomodulatory monoclonal antibodies [37], such as those against CTLA-4, PD-1 and PD-L1 already in clinical use, to remove immunosuppressive mechanisms (“immune checkpoints”) that hamper the induction of effective anti-tumor immune responses [38]. We expect that the analysis of current and forthcoming therapeutic trials will reveal which advances in the field of therapeutic vaccines will be applicable to prophylactic vaccines.

Successful proof-of-principle preclinical studies outlined in the previous sections have opened the way to a few pioneering clinical trials which demonstrate that immunoprevention is indeed feasible in a variety of human conditions at risk of cancer development [30, 39]. It must be kept in mind that cancer prevention trials entail specific hurdles in comparison with therapeutic trials: even in populations at risk, tumor onset is relatively rare, hence large number of volunteers need to be recruited, furthermore, when the subjects harbor preneoplastic or early neoplastic lesions, spontaneous regression is common, even in the absence of any treatment, mandating the need for controlled trials.

Dr. Olivera J. Finn and Robert E. Schoen in Pittsburgh are investigating anti-MUC1 vaccines for the prevention of colorectal carcinogenesis. A pilot clinical trial assessed vaccine immunogenicity in patients with intestinal polyps [40, 41], paving the way to a currently ongoing trial (ClinicalTrials.gov identifier NCT01720836) that aims at preventing polyp onset in tumor-free individuals who previously had a polyp removed. Immunological studies of patients enrolled in the pilot trial revealed that vaccination elicited tumor-specific, cytotoxic anti-MUC1 antibodies [41], thus providing a human counterpart of the preventive mechanisms found in mouse studies of primary immunoprevention (*see above*, Cancer immunoprevention in mice).

The introduction of prophylactic HPV vaccination of girls should not obscure the fact that millions of adult women worldwide harbor a chronic HPV infection that natural immune responses were unable to eradicate, exposing them to a sizeable risk of cervical carcinoma [39]. Prophylactic HPV vaccines, directed against late (L) HPV proteins, lack therapeutic activity against chronic HPV infections [42], thus underlining the need for therapeutic vaccines targeting early (E) HPV proteins [43–45]. One such vaccine, made of electroporated plasmids encoding HPV16/18 oncogenes E6 and E7, increased the occurrence of cervical intraepithelial neoplasia (CIN) regression and of HPV clearance in women harboring HPV-positive CIN 2 or CIN 3 [45].

In mammary carcinogenesis the most obvious target for cancer immunoprevention is HER-2. A neoadjuvant clinical trial conducted in women with HER-2-positive ductal carcinoma in situ (DCIS) undergoing resection within 4–6 weeks showed that the administration of autologous dendritic cells pulsed with HER-2 peptides in vitro elicited anti-HER-2 immune responses in most patients. At surgery, one fourth of all patients had complete tumor regression, the best results was in the ER-negative group, with 38% of complete tumor regressions [46]. Other ongoing vaccination trials are testing an immunodominant HER-2 peptide (nelipepimut-S, also known as E75) in combination with granulocyte-macrophage colony stimulating factor either in a neoadjuvant setting against DCIS (ClinicalTrials.gov NCT02636582) or to prevent the development of metastases after conventional therapy in more advanced, node-positive patients (ClinicalTrials.gov NCT01479244).

A further area in which immunoprevention is already being tested in the clinical arena is that of hematopoietic diseases at risk of progression. After an early clinical trial as single agent [47] a multiepitopic peptide vaccine is being tested in patients with smoldering myeloma in combination with other therapeutic agents to prevent progression to multiple myeloma (ClinicalTrials.gov NCT02886065).

## Conclusions and perspectives

The results of countless mouse studies have demonstrated that the risk of cancer development can be significantly reduced by appropriate treatments that enhance immune defenses.

Primary cancer immunoprevention in healthy humans is currently restricted to tumors related to infectious agents, such as HBV and HPV, which cause about one-sixth of the whole tumor burden. Primary prevention of tumors unrelated to infectious agents is presently unfeasible in the general human population, mainly because it would require vaccines completely devoid of significant toxicities that should confer long-term protection against the risk of a wide spectrum of tumors histotypes. Some hope in this direction comes from the possibility to elicit immune responses against the products of some common mutations in cancer genes, such as RAS or dominant negative p53 [48, 49], but proof-of-principle results in humans are still lacking.

A series of pioneering clinical trials have shown that immunoprevention can be successfully applied to selected human groups in which the risk of a specific tumor type is much higher than in the general population.

Future developments of these concepts might lead to a widespread implementation of cancer immunoprevention, because epidemiological, molecular and genetic studies of the past 100 years have uncovered a huge number of human beings with specific cancer risks, including individuals previously exposed to potent carcinogens, such as asbestos workers or tobacco smokers; patients with preneoplastic or early neoplastic conditions at risk of progression; individuals with genetic predispositions, like microsatellite instability or BRCA mutations.

Finally, most lifestyles (e.g. diet, physical activity) and chemopreventive treatments conducive to reductions in cancer risk also seem to have beneficial effects on the immune system [50], leading to the prediction that complex preventive regimes combining behavioral, chemopreventive and immunopreventive components could have additive or synergistic effects.

## Abbreviations

AIDS: Acquired immune deficiency syndrome
CIN: Cervical intraepithelial neoplasia
CTL: Cytotoxic T lymphocytes
HBV: Hepatitis B virus
HPV: Human papillomavirus
